# Redox Proteomics Changes in the Fungal Pathogen *Trichosporon asahii* on Arsenic Exposure: Identification of Protein Responses to Metal-Induced Oxidative Stress in an Environmentally-Sampled Isolate

**DOI:** 10.1371/journal.pone.0102340

**Published:** 2014-07-25

**Authors:** Sidra Ilyas, Abdul Rehman, Ana Coelho Varela, David Sheehan

**Affiliations:** 1 Dept. Of Microbiology and Molecular Genetics, University of the Punjab, Quaid-e-Azam Campus, Lahore, Pakistan; 2 Instituto de Tecnologia Química e Biológica, Universidade Nova de Lisboa, Oeiras, Portugal; 3 Environmental Research Institute and School of Biochemistry and Cell Biology, University College Cork, Cork, Ireland; Auburn University, United States of America

## Abstract

*Trichosporon asahii* is a yeast pathogen implicated in opportunistic infections. Cultures of an isolate collected from industrial wastewater were exposed for 2 days to 100 mg/L sodium arsenite (NaAsO_2_) and cadmium (CdCl_2_). Both metals reduced glutathione transferase (GST) activity but had no effect on superoxide dismutase or catalase. NaAsO_2_ exposure increased glutathione reductase activity while CdCl_2_ had no effect. Protein thiols were labeled with 5-iodoacetamido fluorescein followed by one dimensional electrophoresis which revealed extensive protein thiol oxidation in response to CdCl_2_ treatment but thiol reduction in response to NaAsO_2_. Two dimensional electrophoresis analyses showed that the intensity of some protein spots was enhanced on treatment as judged by SameSpots image analysis software. In addition, some spots showed decreased IAF fluorescence suggesting thiol oxidation. Selected spots were excised and tryptic digested for identification by MALDI-TOF/TOF MS. Twenty unique *T. asahii* proteins were identified of which the following proteins were up-regulated in response to NaAsO_2_: 3-isopropylmalate dehydrogenase, phospholipase B, alanine-glyoxylate aminotransferase, ATP synthase alpha chain, 20S proteasome beta-type subunit Pre3p and the hypothetical proteins A1Q1_08001, A1Q2_03020, A1Q1_06950, A1Q1_06913. In addition, the following showed decreased thiol-associated fluorescence consistent with thiol oxidation; aconitase; aldehyde reductase I; phosphoglycerate kinase; translation elongation factor 2; heat shock protein 70 and hypothetical protein A1Q2_04745. Some proteins showed both increase in abundance coupled with decrease in IAF fluorescence; 3-hydroxyisobutyryl- CoA hydrolase; homoserine dehydrogenase Hom6 and hypothetical proteins A1Q2_03020 and A1Q1_00754. Targets implicated in redox response included 10 unique metabolic enzymes, heat shock proteins, a component of the 20S proteasome and translation elongation factor 2. These data suggest extensive proteomic alterations in response to metal-induced oxidative stress in *T. asahii*. Amino acid metabolism, protein folding and degradation are principally affected.

## Introduction

*Trichosporon asahii* is an emerging yeast pathogen, which is increasingly reported in nosocomial infections, in fatal opportunistic infections and in summer-type hypersensitivity pneumonitis [1]–[3]. It occupies a wide variety of environmental niches [3]–[6]. Some yeasts such as *Trichoderma spp.* and *Rhodotorula spp.* are known to be able to accumulate and survive exposure to heavy metals [7]–[10]. Yeasts have evolved impressive antioxidant defense systems, which include glutathione transferase (GST) [11], glutathione reductase (GR), catalase (CAT) and superoxide dismutase (SOD) activities. Despite the known metal-resistance potential of many yeasts [7]–[10]; [12], for most species only limited information is available about proteomic changes attendant on metal accumulation. We are interested in yeasts of pollutant-rich wastewaters from the textile industry and serendipitously cultured an isolate of *T. asahii* from this source. Because of its metal-tolerance, growing clinical importance [1]–[3] and the recent availability of its genome [13], [14], we felt that this isolate might be an attractive and topical target for proteomic profiling of heavy metal response systems in *T. asahii*.

Molecular oxygen is essential for aerobic life-forms but it can also produce highly toxic reactive oxygen species (ROS) [15]–[18]. Heavy metals (e.g. Cd) and metalloids (e.g. As), which are common environmental pollutants can contribute to ROS generation in yeast cells [19]–[23]. Proteins absorb ∼70% of ROS causing reversible and irreversible covalent changes to amino acid side-chains of proteins that can complicate the proteome [17], [18], [24]–[26]. For example, the cysteine thiol group (-SH) can become reversibly oxidized to sulphenic acid (-SOH) or disulphides (-S–S- or -S–S–glutathione) and irreversibly modified to sulphinic (-SO_2_H) or sulphonic (-SO_3_H) acids [18], [27]. Amino acid side chains can also be modified forming aldehyde or ketone groups (protein carbonylation), which can lead to protein aggregation, inactivation or degradation [28], [29]. These effects have been extensively investigated in systems such as bacteria, yeast, mammalian tissues and cell cultures [29]–[32]. Environmental pollutants such as metals, pesticides, endocrine disruptors and nanomaterials are known to trigger oxidative stress in a range of organisms [33]–[36]. This may be due to direct production of ROS from reactions such as the Fenton reaction [18], damage to mitochondria leading to leakage of ROS [17] or changes in redox signalling resulting from stress pathways [36]. Such oxidation can be detected by measuring lipid peroxidation or oxidation of proteins. Recently, redox proteomics has proved an especially robust approach to detection of oxidative stress effects on proteins in environmental pollution scenarios [37]–[39].

Environmental pollutants such as Cd and As are considered highly toxic as they have a high affinity for proteins and can alter protein structure and function [40], [41]. They can either bind directly to protein -SH groups or indirectly generate oxidative modifications within yeast cells [42]–[44], which cause changes in proteins as sulphur-containing residues are so susceptible to oxidation [45]. In addition, both Cd and As are also known to cause protein carbonylation [46], [22]. The present study probed changes in antioxidant defense proteins of *T. asahii* as a primary response to oxidative stress (OS) experienced in response to CdCl_2_ and NaAsO_2_.

## Materials and Methods

### Chemicals and Reagents

Acrylamide, ammonium sulphate, 1-chloro-2,4-dinitrobenzene (CDNB), dimethyl sulphoxide (DMSO), dithiothreitol (DTT), ethylenediamine-tetraacetic acid (EDTA), ferrous sulphate, hydrogen peroxide, iodoacetamide, nicotinamide adenine dinucleotide phosphate NADP^+^, NADPH (reduced form of NADP^+^), nitro blue tetrazolium (NBT), oxidized glutathione (GSSG), phenylmethylsulphonyl fluoride (PMSF), reduced glutathione (GSH), sodium dodecyl sulphate (SDS), *N*,*N*,*N_*,*N_* tetramethylethylenediamine (TEMED), thiourea, trichloroacetic acid (TCA), Tris–HCl, urea were purchased from Sigma–Aldrich Corporation (USA). All general electrophoresis reagents were obtained from GE Healthcare. Metal ions used for producing oxidative stress were cadmium chloride (CdCl_2_) and sodium arsenite (NaAsO_2_).

### Culture and experimental conditions

*T. asahii* was collected from metal-containing effluents in sterilized glass containers and yeast cells were sub-cultured on YPD [2% (w/v) glucose, 1% (w/v) yeast extract, 2% (w/v) bacto-peptone and 2% (w/v) agar (pH 6.5)] agar plates. Biomass was grown to log phase on a liquid salt medium containing: 1% (w/v) glucose, 0.1% (w/v) (NH_4_)_2_SO_4_, 0.015% (w/v) KH_2_PO_4_, 0.01% (w/v) K_2_HPO_4_, 0.01% (w/v) MgSO_4_·7H_2_O, 0.0026% (w/v) FeSO_4_, 0.0086% (w/v) CaCl_2_ (pH 7.0–7.2) with constant shaking incubation (120 rpm) at 30°C. After 24 hours, NaAsO_2_ and CdCl_2_ were added to yeast cultures to a final concentration of 100mg/L and cells were grown for a further 2 days. Controls were treated identically but without heavy metal exposure.

### Survival to metal exposure

Cells were cultured on YPD agar plates at 30C in increasing concentrations of NaAsO_2_ and CdCl_2_ and minimum inhibitory concentration assessed [47]. The initial metal concentration used was 1 mM prepared from a 1 M metal stock solution. Grown yeast cells were subsequently transferred at a given concentration to next concentration and maximum resistance was evaluated until *T. ashaii* was unable to grow as colonies on metal-containing agar plates. Any colour changes of colonies or cultures in response to metal exposure were carefully noted.

### Enzyme preparation

Approximately 1 g yeast cells (wet weight) was crushed in pestle and mortar to a fine powder in liquid nitrogen and suspended in 2 ml of buffer containing 10 mM Tris-Cl buffer (pH 7.2), 1 mM EDTA, 0.5 M sucrose, 0.15 M KCl and 1 mM PMSF. Homogenates were centrifuged at 11,000×g for 15 min. (Sorvall RC 5C plus) and supernatants were immediately stored in aliquots at −80°C until required for further analysis. Protein concentration from each cell free sample extract was quantified by the method of Bradford [48] using bovine serum albumin (BSA) as a standard. All operations were carried out at 4°C.

### Enzyme activities

Biochemical measurements of glutathione-dependent enzymes (GST and GR) and antioxidant (SOD and CAT) were conducted using a spectrophotometer/microplate reader and activities were measured separately in response to metal ion treatment. Each enzymatic activity was determined in quadruplicate for each sample and data reported are means +/− standard deviations of three independent experiments. One unit (U) of enzyme activity is defined as the amount consuming 1 µM of substrate or generating 1 µM product/min.

### Glutathione transferase

GST was determined by the method of Habig *et al.*, [49]. The reaction mixture contained substrate for glutathione (GSH); 1 mM 1-chloro-2, 4- dinitrobenzene (CNDB), 1 mM reduced glutathione (GSH) in 150 mM phosphate buffer (pH 6.5) and enzyme extract. The formation of *S*-2, 4-dinitrophenyl glutathione conjugate was followed as an increase in absorbance at 340 nm (every 20 or 30 s) for 5 min at 25°C.

### Glutathione reductase

GR was determined by the method of Carlberg and Mannervik [50]. The reaction volume of 0.2 ml contained 0.48 M phosphate buffer (pH 7.2), 1.1 mM EDTA with 8.5 mM oxidised glutathione (GSSG) and extract. The reaction mixture was equilibrated at 25°C before adding freshly-prepared 0.65 mM NADPH. Absorbance was read at 340 nm for 5 min.

### Catalase

CAT activities were determined according to Beers and Sizer [51] based on disappearance of H_2_O_2_ recorded at 240 nm for 2–3 minutes at 25°C. The reaction mixture contained 30% H_2_O_2_ in potassium phosphate buffer (50 mM, pH 7.0), 1.9 ml reagent grade water and crude enzyme source.

### Superoxide dismutase assay

SOD activity was determined by an indirect assay involving the inhibition of NBT reduction [52]. The reaction mixture contained 62.5 mM sodium phosphate buffer (pH 7.4), 0.125 mM EDTA, 83.3 µM NBT, 150 µM NADH and 16.5 µM PMS. Reduction of NBT was monitored at 25°C as an absorbance increase at 560 nm (every 20 or 30 s) for 5 min. One U of SOD activity is defined as that amount of enzyme causing 50% inhibition of NBT.

### One and two dimensional gel electrophoresis

One dimensional electrophoresis (1-DE) was performed in 14% (w/v) polyacrylamide gels [53]. Proteins from three independent cultures were pre-labeled with IAF prior to electrophoresis [54]. Tagged protein extracts were precipitated in 10% TCA followed by centrifugation for 3 min at 10,000×g. Supernatants were discarded and pellets solubilized in sample buffer. Marker (4 µl) and samples (20 µl) were heated at 95°C prior to loading for 3 min. Fluorescence images of proteins were captured on a Typhoon scanner (model 9410, GE Healthcare) using 450 PMT and intensity of fluorescence for each lane was calculated using ImageQuant software.

For 2-DE, protein thiols in protein extracts were pre-labeled with IAF prior to electrophoresis [54]. Tagged protein extracts (150 µg) were precipitated in 10% trichloroacetic acid (TCA), washed in cold acetone and dissolved in rehydration buffer containing 5 M urea, 2 M thiourea, 2% (w/v) CHAPS, 1% (w/v) De- Streak reagent (GE Healthcare), 4% (w/v) carrier ampholyte (Pharmalyte 3–10; Amersham-Pharmacia Biotech, Little Chalfont, Bucks. UK) and a trace amount of bromophenol blue. A final volume of 125 µl was incubated overnight on 7-cm IPG dry strips (pH 3–10; BioRad, Hercules, CA, USA) and focused in a Protean IEF Cell (BioRad). IEF was initiated with linear voltage increases: 250 V for 30 min, 4000 V for 2 h and then up to 40,000 Vh for 7 cm IPG strips. IPG strips were equilibrated twice, first in DTT (0.5% w/v) followed by iodoacetamide (2.5% w/v) for 20 min. with gentle agitation on rocker in equilibration buffer [6 M urea, 0.375 M Tris-HCl (pH 8.8), 20% (v/v) glycerol, 2% (w/v) SDS]. After equilibration, IPG strips were rinsed with 1X running buffer and loaded on 14% (w/v) polyacrylamide gels along with 4 µl of standard proteins on filter paper covered with molten agarose (0.5% w/v) containing a trace of bromophenol blue. Electrophoresis was performed at 90 V for 30 min. followed by a constant voltage (120 V) using a mini PAGE system (Atto, Tokyo, Japan) until the dye front reached the end of the gel cassette. Gels were washed three times with Millipore water and fluorescence images of proteins captured on a Typhoon scanner (model 9410, GE Healthcare) using 450 PMT and intensity of fluorescence measured. Protein expression profiles (PEPs) in analytical gels were visualized by colloidal coomassie G-250 (Bio-Rad) staining [55].

### Image analysis of two dimensional gels

Gels were scanned for fluorescence in a Model 9410 Typhoon scanner (Amersham-Pharmacia Biosciences, UK) and then stained with colloidal coomassie [55] before scanning in a calibrated GS-800 scanner (BioRad, USA). Image analysis was performed on either fluorescence or coomassie-stained images using Progenesis SameSpots software (Nonlinear Dynamics, Newcastle-Upon-Tyne, Tyne and Wear, UK) according to the manufacturer's instructions. The images were automatically aligned and then edited manually as per the SameSpots workflow which enabled close to 100% spot matching. All images successfully passed the SameSpots quality assurance step. The SameSpots software uses powerful statistical analysis and arrives at conclusions by principal component analysis (PCA), correlation analysis, power analysis, and q-values to explore the data. Differentially expressed proteins were selected visually after this analysis. These spots were either present or absent in control or treatments. Spots were considered of interest when displaying <1.5-fold> difference between test and control samples as well as p<0.05 in ANOVA analysis.

### Identification of proteins by MALDI-TOF/MS

Proteins were manually picked from 2DE separations, lightly stained with colloidal coomassie. Following in-gel tryptic digestion, extracted peptides were loaded onto a R2 micro-column (RP-C18 equivalent) where they were desalted, concentrated and eluted directly onto a MALDI plate using α-cyano-4-hydroxycinnamic acid (CHCA) as the matrix solution in 50% (v/v) acetonitrile and 5% (v/v) formic acid. Mass spectra of the peptides were acquired in positive reflectron MS and MS/MS modes using a MALDI-TOF/TOF MS instrument (4800*plus* MALDI TOF/TOF analyzer) with an exclusion list of the trypsin autolysis peaks (842.51, 1045.56, 2211.11 and 2225.12). The collected MS and MS/MS spectra were analysed in combined mode by Mascot search engine (version 2.2; Matrix Science, Boston, MA) and the NCBI database restricted to 50 ppm peptide mass tolerance for the parent ions, an error of 0.3 Da for the fragments, one missed cleavage in peptide masses, and carbamidomethylation of Cys and oxidation of Met as fixed and variable amino acid modifications, respectively. No taxonomy restrictions were applied. The identified proteins were only considered if a MASCOT score above 95% confidence was obtained (p<0.05) and at least one peptide was identified with a score above 95% confidence (p<0.05). This analysis was conducted by the Analytical Services Unit, Instituto de Tecnologia Química e Biológica (ITQB), New University of Lisbon, Lisbon, Portugal. Spot identifications and raw MS data were uploaded to the ProteomeXchange website (http://www.proteomexchange.org) and allocated the accession number 1-20140522-114745.

### Statistical analyses

All data were obtained from a minimum of three independent experiments and calculated as means plus/minus standard deviations (±SD). Statistical analyses on enzyme specific activity data were performed using paired Student's *t*-test. The data obtained from protein gel images were tested for normality prior to any analysis and significance testing level was set at 0.05. Principal component analysis (PCA) and analysis of variance (one way ANOVA) was performed for comparison and assessment of statistical significant expression and redox variation between treatments and control.

## Results and Discussion

### Identification and culture conditions

Whilst screening for pollutant-resistant environmental fungi, a metal-resistant yeast was serendipitously isolated from wastewater of a textile plant in Lahore, Pakistan, [56], [57]. Nucleotide sequences were determined at the Center of Excellence in Molecular Biology, Lahore, Pakistan and identity with T. asahii confirmed using the basic local alignment search tool (). The isolate was maintained on YPD agar plates and grew well in a liquid salt medium in a 1% (w/v) glucose carbon source. Cultures survived well in 100 mg/L NaAsO_2_ and CdCl_2_, which corresponded to doses of 680 µM (Cd) and 770 µM (As). *T. asahii* is known to be widespread in the environment, presumably facilitating its role as an opportunistic pathogen [3]–[6], but its survival in a pollutant-rich industrial wastewater led us to explore the biochemical basis of its unexpected metal-resistance.

### Tolerance to metal exposure

**Figure 1:**
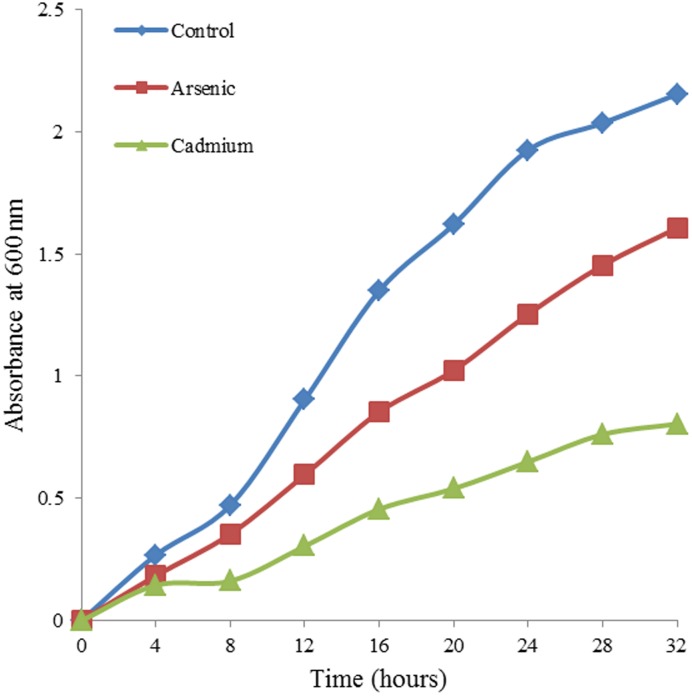
Growth curves for *T. asahii* cultures grown in the presence of 100 mg/L CdCl2 (green) and NaAsO2 (red).

Metal tolerance of *T. asahii* isolates used in this study was assessed in both NaAsO_2_ and CdCl_2_ [57]. Cells tolerated NaAsO_2_ up to 30 mM and CdCl_2_ up to 10 mM. Biomass (Table 1) and growth rates (Fig. 1) were only modestly decreased when exposed to 100 mg/L NaAsO_2_ and CdCl_2_.

**Table 1:** Fresh weight of *T. asahii* yeast environmental isolate grown in minimal salt media supplemented with and without heavy metals.

Yeast isolate *T. asahii*	Fresh weight expressed as %			
	Sample 1	Sample 2	Sample 3	Mean ± SD
Control (without heavy metal)	100	100	100	100
Cadmium Chloride (CdCl2)	71	96	78	82±12.9
Sodium Arsenite (NaAsO_2_)	91	79	92	87.3±7.2

Three biological and four technical replicates were used for further study and an equal gram weight was taken for protein extraction.

### Enzyme activity responses to cadmium and arsenite

Culturing of *T. asahii* cells in the presence of CdCl_2_ caused noticeable phenotypic changes suggesting a profound biological response to this metal. A decrease in fresh weight and biomass content was noted (Table 1). Cells also seemed to alter their cell wall structure and composition as they became noticeably much more difficult to lyse. Cadmium and other metals can sorb to cell walls of plants, algae, fungi and bacteria [56], so it is possible that this explains the change in cell wall characteristics observed here. In addition, colonies changed colour to brown from off-white and cell texture was also noted to be altered. A change in colour in response to metals has previously been noticed for *Trichsporon sp* grown in the presence of lead [57].

**Figure 2:**
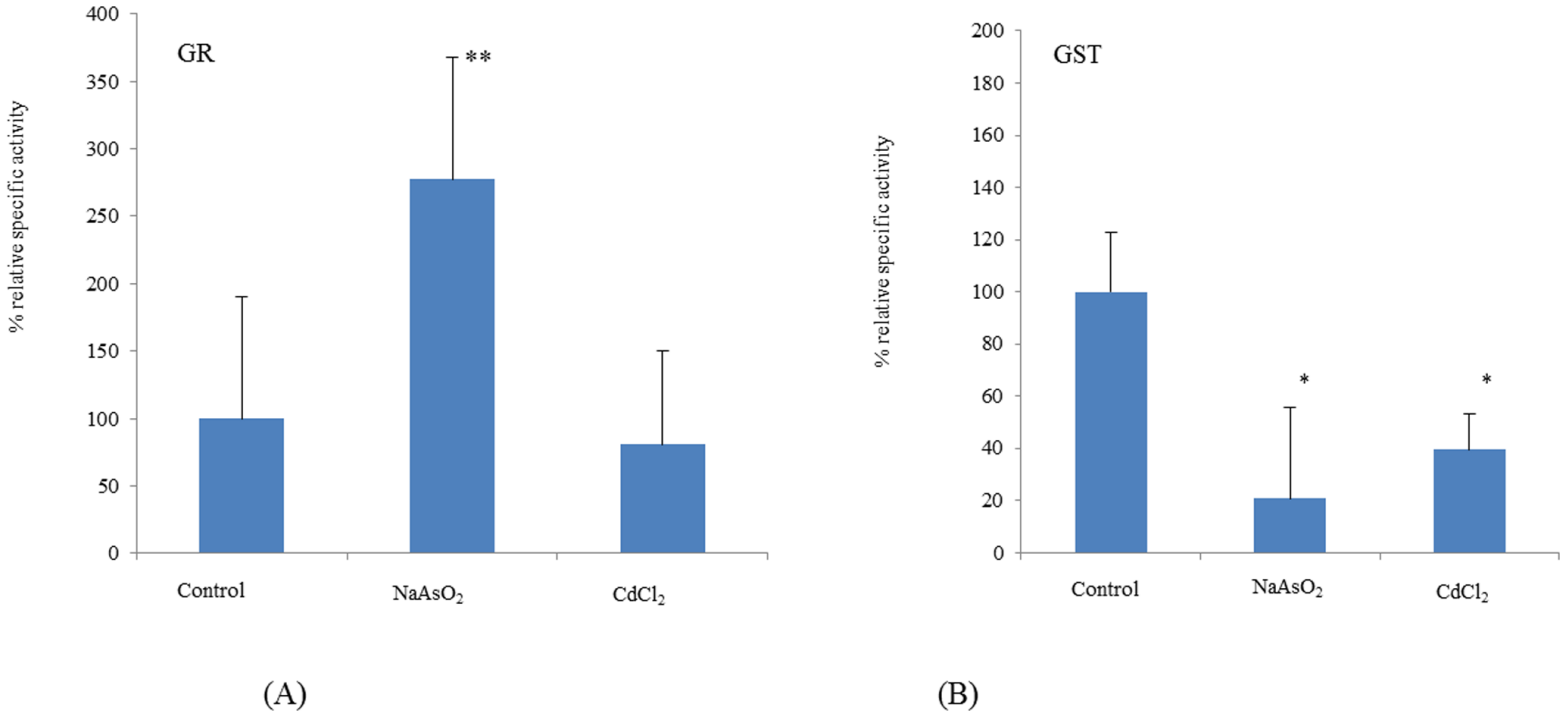
Relative specific activities for GR (panel A) and GST (panel B) in *T. asahii* cultures exposed to CdCl_2_ and NaAsO_2_. Values are expressed as mean of SD and experiments were performed in triplicate (*p<0.05; **p<0.1 when compared with controls).

**Figure 3:**
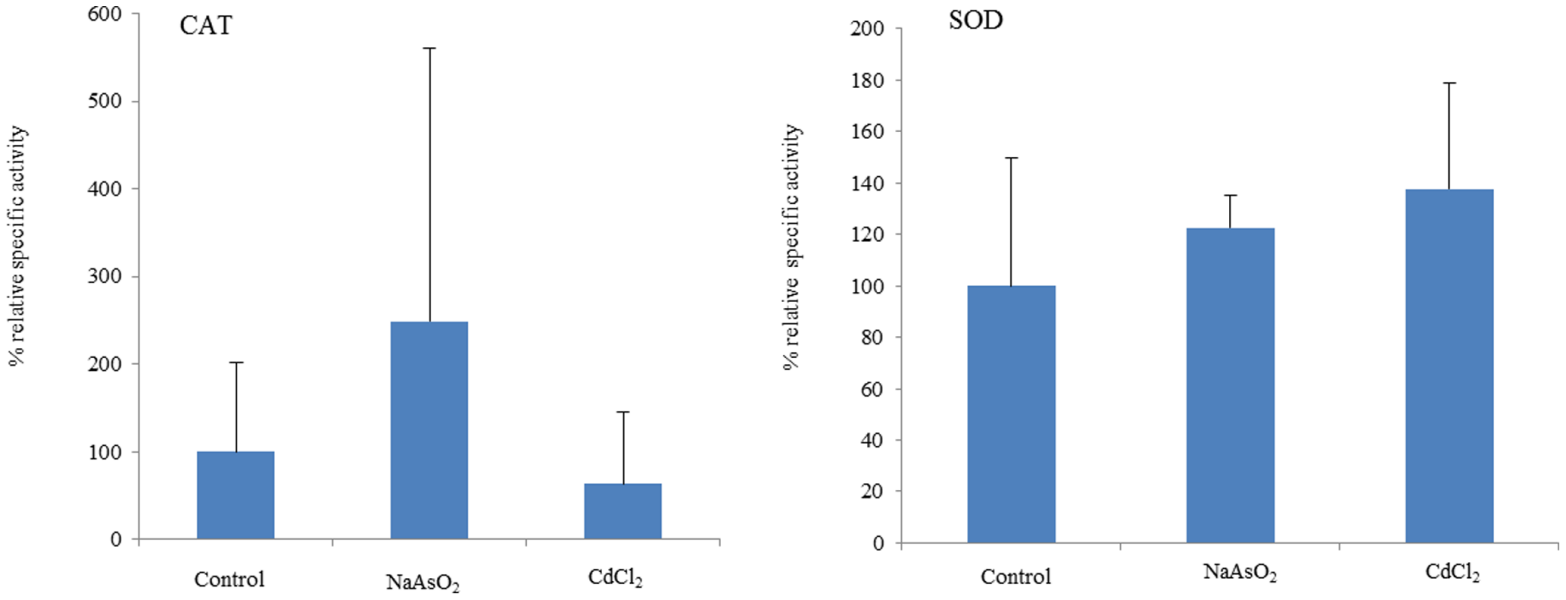
Relative specific activities for CAT (panel A) and SOD (panel B) in *T. asahii* extracts from yeast cultured in the presence of CdCl_2_ and NaAsO_2_. Values are expressed as mean of SD and experiments were performed in triplicate.

It was reasoned that detoxification and antioxidant enzymes such as GST, GR, SOD and CAT might contribute to metal-resistance by analogy with other yeasts [7]–[10], [58], [59]. Exposure to both CdCl_2_ and NaAsO_2_ strongly reduced GST activity (Fig. 2) but had no significant effect on SOD or CAT (Fig. 3). Whilst NaAsO_2_ exposure caused increased GR activity, CdCl_2_ had no effect on this enzyme (Fig. 2) indicating a level of specificity in terms of metal effects on this panel of enzymes.

These data suggest that *T. asahii* possesses adequate CAT and SOD activity to cope with metal-related ROS and did not need to induce higher levels of these antioxidant enzymes. However, enhanced GR on exposure to NaAsO_2_ indicates that *T. asahii* has a requirement for more GSH on exposure to this metal which was not the case for CdCl_2_. *Saccharomyces cerevisiae* mutants for yap1 have previously been reported to show both increased levels of carbonylated proteins and decreased GSH:GSSG ratio on exposure to arsenic [22] which is consistent with our observed arsenic-related effects on GR. Both metals triggered a large decrease in GST activity, which may indicate inactivation of this enzyme by exposure to ROS.

### Protein oxidation effects revealed by one-dimensional electrophoresis

**Figure 4:**
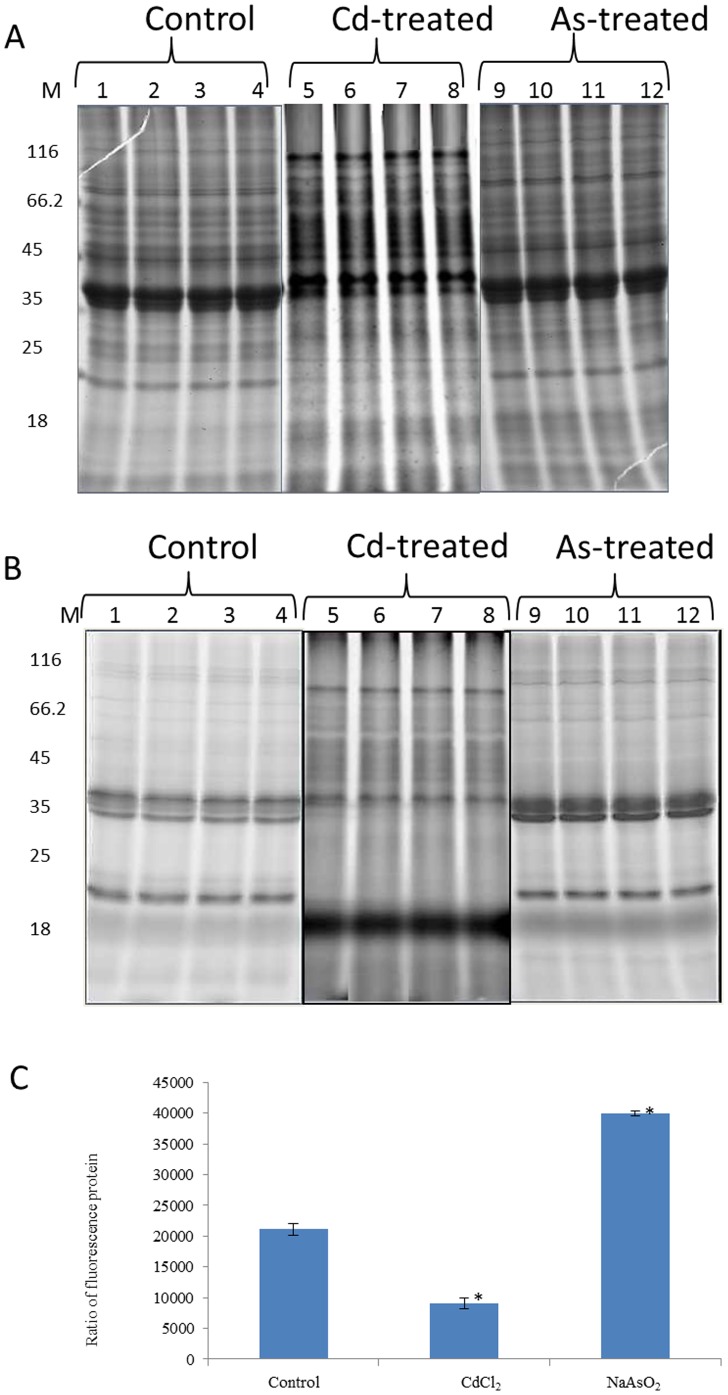
1DE analysis of IAF-labelled *T. asahii* proteins. Proteins (150 µg) extracted from *T. asahii* were labelled with 0.2 mM IAF and subjected to 1DE. The gels were scanned for IAF fluorescence in a Typhoon scanner followed by staining in colloidal coomassie. Panel A shows coomassie-stained gels whilst panel B is the corresponding IAF fluorescence image. Changes in total spot intensity between treated and untreated cells were quantified by Image Quant software analysis (Panel C).

Oxidation of proteins occurs commonly in metal-induced OS [18]–[24]. The thiol group of cysteine is notoriously susceptible to such oxidation [17], [18]. Analysis of the *T. asahii* proteomic response to CdCl_2_ and NaAsO_2_ could be potentially important for understanding the mechanism of metal-resistance. Thiol groups of proteins were tagged with IAF (which does not label oxidized variants of protein thiols) and proteins separated by 1-DE [53]. Total IAF-associated fluorescence was measured in each electrophoresis lane for control and metal-exposed extracts prior to protein staining. A ratio of fluorescence to colloidal coomassie staining intensity was then calculated (Fig. 4). This revealed that CdCl_2_ caused a strong reduction of total protein thiols while NaAsO_2_ caused an increase of thiol groups. This suggests that CdCl_2_ causes OS evident at the protein level while NaAsO_2_ had a protective effect to OS, which is consistent with the observed enhanced GR activity (Fig. 2). A key contribution of GSH is to maintain protein thiols in their reduced state and elevated GR activity would be expected to contribute strongly to this. A recent report in *S. cerevisiae* showed that arsenite challenge increased GSH biosynthesis with attendant increase in GR expression [60].

### Protein oxidation effects by two-dimensional electrophoresis

**Figure 5:**
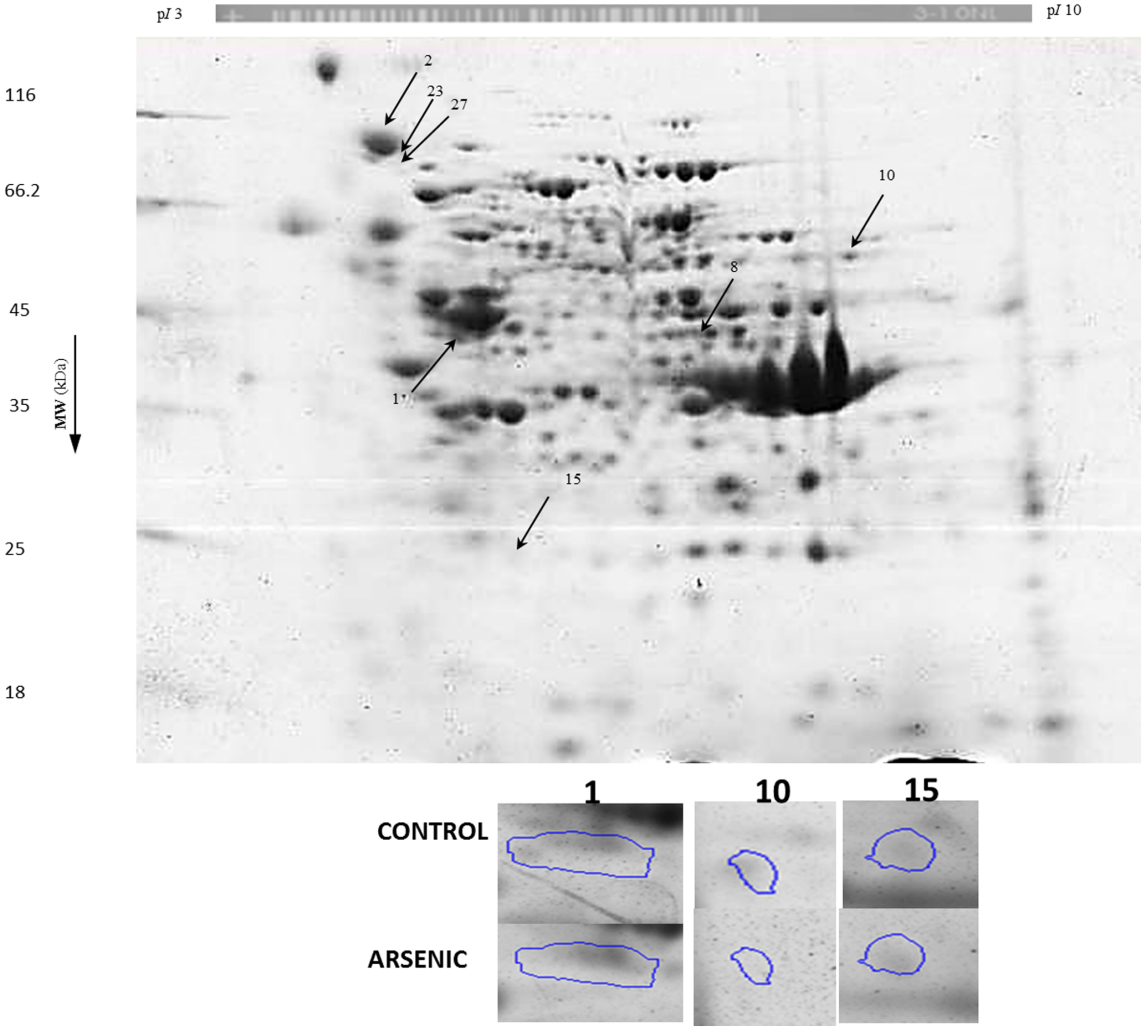
2DE analysis of proteins from *T. asahii* stained with colloidal coomassie. Following fluorescence scanning, gels were stained with colloidal coomassie brilliant blue. Increase or decrease of protein spot intensity was estimated by Progenesis Samespot analysis software and images were normalized prior to applying any statistics. Spots excised for identification are indicated by arrows. Circles represent spots showing variation both in protein expression and in thiol oxidation (*see* Table 4). Inset: Zoom boxes are shown for spots.

**Figure 6:**
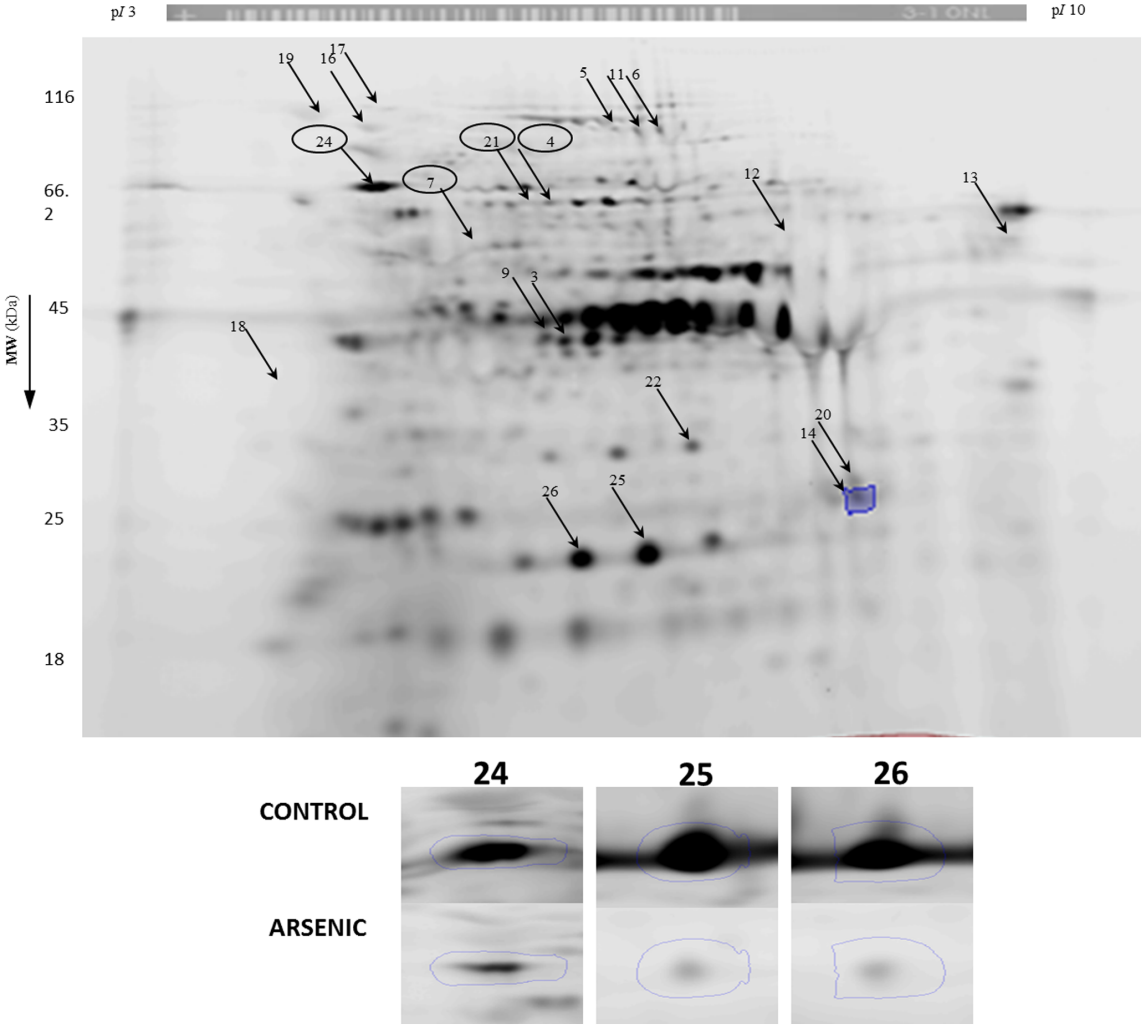
2DE analysis of proteins from *T. asahii* labeled with IAF at protein thiols. Prior to 2DE, protein thiols were labeled with IAF. Gels were scanned for IAF-associated fluorescence and images were analysed by Progenesis Samespot analysis software. Images were normalized prior to applying any statistics. Spots showing significant (p<0.05) decrease in IAF-associated fluorescence were excised for identification and are indicated by arrows (*see* Table 2). Inset: Zoom boxes are shown for spots.

Because of the effects on cell wall rigidity, it was not possible to extract sufficient protein from CdCl_2_-treated *T. asahii* for 2-DE analysis in this study. Therefore, protein expression signatures (PES) were compared only between control and NaAsO_2_ –treated cells (Fig. 5). This comparison matched 1075 spots and revealed up-/down-regulation of certain spots quantified by SameSpots image analysis software in either colloidal coomassie-stained gels (Fig. 5) or IAF fluorescence intensity (Fig. 6) with p<0.05 and intensity changes <1.5-fold>. In total, based on either changes in colloidal coomassie staining intensity or in IAF-associated fluorescence, 40 spots were excised for in-gel tryptic digestion and identification by MALDI-TOF/TOF MS. Of these, 31 spots were successfully identified (see arrows in Figs. 5 and 6) and 26 of these matched well to the two recently-released genomes for *T. asahii* for strains CBS 8904 [13] and CBS 2479 [14] (Table 2). The sole non-*T. asahii* protein spot (spot no. 14) matched to an endoribonuclease of *Aeromonas hydrophila*, a heterotrophic, gram-negative bacterium known to inhabit water in areas of warm climate which sometimes causes necrotizing fasciitis in humans. It is possible that this bacterium contaminated our *T. asahii* cultures. Eighteen spots were identified as containing *T. asahii* proteins of known function whilst 8 were found to contain hypothetical proteins. A striking number of metabolic enzymes (12 of 18 spots representing 10 unique enzymes) were identified as well as heat shock proteins (4 of 18 spots representing 3 unique proteins). The two other proteins were Pre3p, a component of the 20S proteasome, and the translation elongation factor 2 (Table 2).

**Table 2:** Identification of *T. asahii* yeast proteins.

Spot	Identified Protein	Organism	Accession number d	MW m	Protein Score^a^/Confidence Interval (%)	Ion Score/Confidence Interval (%)	Sequence Coverage (%)	No. of MS/MS patterns assigned to peptides^b^	P-value	Fold change
*Metabolic enzymes (energy and respiration)*										
1	3-Isopropylmalate dehydrogenase	*Trichosporon asahii* var. asahii CBS 8904	gi|406698071	53569	694/100	639/100	44	6	6.43E-04	1.6
2	Phospholipase B	*Trichosporon asahii* var. asahii CBS 2479	gi|401884039	71965	870/100	837/100	27	7	0.002	1.6
3	Malate dehydrogenase	*Trichosporon asahii* var. asahii CBS 2479	gi|401884576	35270	1190/100	1133/100	67	8	0.005	−2
4	3-Hydroxyisobutyryl- CoA hydrolase	*Trichosporon asahii* var. asahii CBS 8904	gi|406696953	57118	1190/100	1073/100	61	8	0.005	−2.2
5	Aconitase	*Trichosporon asahii* var. asahii CBS 8904	gi|406694125	85654	1320/100	1228/100	36	9	0.007	−2.5
6	Aconitase	*Trichosporon asahii* var. asahii CBS 8904	gi|406694125	85654	1320/100	1228/100	36	9	0.008	−1.6
7	Homoserine dehydrogenase Hom6	*Trichosporon asahii* var. asahii CBS 8904	gi|406695321	40376	338/100	303/100	28	2	0.017	−1.7
8	Alanine-glyoxylate aminotransferase	Trichosporon asahii var. asahii CBS 2479	gi|401885827	51027	891/100	815/100	45	7	0.017	2
9	Aldehyde reductase I (Alcohol dehydrogenase)	*Trichosporon asahii* var. asahii CBS 2479	gi|401886852	64647	1560/100	1479/100	50	10	0.02	−1.9
10	ATP synthase alpha chain, precursor	*Trichosporon asahii* var. asahii CBS 2479	gi|401889058	58090	1170/100	1068/100	47	9	0.023	1.7
11	Aconitase	*Trichosporon asahii* var. asahii CBS 8904	gi|406694125/	85654	799/100	712/100	35	5	0.015	−2
12	Phosphoglycerate kinase	*Trichosporon asahii* var. asahii CBS 8904	gi|406694335	49924	1890/100	1734/100	61	10	0.025	−1.5
*Protein biosynthesis and folding*										
13	Translation elongation factor 2	*Trichosporon asahii* var. asahii CBS 8904	gi|406694343	51124	423/100	397/100	36	4	0.009	−3.4
14	Endoribonuclease LPSP	*Aeromonas hydrophila* subsp. hydrophila ATCC 7966	gi|117619955	13357	199/100	186/100	46	1	4.45E-05	2.3
*Nonfunctional and abnormal protein degradation*										
15	20S Proteasome beta-type subunit, Pre3p	*Trichosporon asahii* var. asahii CBS 8904	gi|406698063	25360	435/100	422/100	33	4	0.036	1.8
*Protein repair and folding*										
16	Heat shock protein	*Trichosporon asahii* var. asahii CBS 2479	gi|401888774	71440	548/100	490/100	39	4	0.002	−3.1
17	Heat shock protein	*Trichosporon asahii* var. asahii CBS 8904	gi|406695586	98932	1200/100	1065/100	42	7	0.019	−3.1
18	Heat shock protein 70	*Trichosporon asahii* var. asahii CBS 2479	gi|401882131	70187	846/100	798/100	34	8	0.021	−4.5
19	Heat shock protein70	*Trichosporon asahii* var. asahii CBS 2479	gi|401882131	70187	1960/100	1704/100	63	11	0.034	−2.1
*Hypothetical Proteins*										
20	Hypothetical protein A1Q1_08001	*Trichosporon asahii* var. asahii CBS 2479	gi|401886770	21217	1310/100	1266/100	53	9	3.44E-05	2.5
21	Hypothetical protein A1Q2_03020	*Trichosporon asahii* var. asahii CBS 8904	gi|406699588	60263	441/100	403/100	35	2	0.011	−2.4
22	Hypothetical protein A1Q1_06950	*Trichosporon asahii* var. asahii CBS 2479	gi|401887835	33117	432/100	399/100	30	3	0.011	1.9
23	Hypothetical protein A1Q1_06913	*Trichosporon asahii* var. asahii CBS 2479	gi|401887891	71289	1460/100	1403/100	38	11	0.011	1.8
24	Hypothetical protein A1Q1_00754	*Trichosporon asahii* var. asahii CBS 2479	gi|401888905	53663	539/100	503/100	42	4	0.013	−1.6
25	Hypothetical protein A1Q2_04745	*Trichosporon asahii* var. asahii CBS 8904	gi|406697616	53697	333/100	333/100	20	2	0.016	−2.1
26	Hypothetical protein A1Q2_04745	*Trichosporon asahii* var. asahii CBS 8904	gi|406697616	53697	341/100	341/100	7	2	0.034	−1.9
27	Hypothetical protein A1Q1_06913	*Trichosporon asahii* var. asahii CBS 2479	gi|401887891	71289	964/100	904/100	38	8	0.041	1.6

Protein thiols were labeled with IAF following exposure to NaAsO_2_ and identified by MALDI-TOF/TOF MS. MW (molecular masses) are expressed in Da. Spots (1–12) showed up-regulated metabolic enzymes; (16–19) were involved in protein synthesis and repair enzymes; (20–31) were hypothetical proteins. Spots taken arbitrarily showed NA p-value and fold-change. These all proved to be hypothetical proteins.

a Protein score probability limit (where P<0.05) was 86.

b Peptides with confidence interval >95% were considered.

d Database accession number obtained from NCBI (top hits shown).

m mass of identified proteins.

Some of the proteins identified here are well-known to be redox-sensitive in *S. cerevisiae*; phosphoglycerate kinase, translation elongation factor 2 and aconitase [61]. In the cyanobacterium *Anabaena* sp. arsenic has been reported to induce phosphoglycerate kinase, ATP synthase alpha subunit and hsp 70 all of which feature in Table 1 [62] while arsenic-challenged *Klebsiella pneumoniae* up-regulates alcohol dehydrogenase, aconitase and hsp 70 and down-regulates malate dehydrogenase [63]. It is noteworthy that 3-isopropylmalate dehydrogenase, alanine-glyoxylate aminotransferase, 3-hydroxyisobutyryl CoA hydrolase and homoserine dehydrogenase are all involved in amino acid metabolism suggesting a strong metabolic effect of NaAsO_2_ on this aspect of cell physiology. Of 53 metabolic proteins identified in misfolded protein aggregates arising from arsenite exposure in *S. cerevisiae*, 27 were associated with amino acid metabolism [64]. Similarly, identification of hsp 70 and a component of the 20S proteasome is consistent with arsenite toxicity being mediated via effects on protein folding in *S. cerevisiae* [64]. Arsenite challenge also causes changes to sulphur metabolism in *S. cerevisiae* leading to increased GSH biosynthesis [60]. Our data suggest that NaAsO_2_ elicits strong effects on key cellular processes such as redox-response, amino acid metabolism, protein folding/mis-folding and protein turnover in *T. asahii*.

More than one third of *T. asahii* spots studied here (8 of 26) were identified as 6 unique hypothetical proteins (Table 2). These commonly comprise 30–40% of microbial genes [65], [66]. There is growing interest in mining microbial genomes to improve annotation of hypothetical proteins by a variety of functional, computational and structure analysis approaches [66], [67]. In the present context, it is evident that the 6 unique hypothetical proteins identified here are indeed expressed at the protein level and they are likely to be of functional significance to *T. asahii* in response to NaAsO_2_. In an effort to ascribe putative functions to these proteins, searches were performed for the six accessions identified in NCBI (http://www.ncbi.nlm.nih.gov/) and putative functional domains were identified in the conserved domain database (CDD) [68]. Full-length sequences were also entered in Pfam to identify protein families by sequence alignment [69]. This analysis is summarized in Table 3. Whilst allocation of function to hypothetical proteins must of necessity be tentative, possible roles for four of the hypothetical proteins can be suggested. A1Q1_08001 contains an N-terminal domain common in NADP(H) dehydrogenases. NADPH is the key source of reducing equivalents for reduction of GSH and induction of NADPH-producing pathways such as the pentose phosphate pathway is commonly observed in oxidative stress [70]. A1Q2_03020 contains an aldehyde dehydrogenase (ADH) domain. ADHs detoxify aldehydes by reducing them to carboxylic acids and are often expressed in stress-response scenarios in which some ADHs form part of phase 2 of detoxification [71]. Both A1Q1_06950 and A1Q1_06913 contain a domain similar to the lactam utilization protein LamB/YcsF family of proteins, which may be involved in hydrolysis of glycosidic bonds. In addition, A1Q1_06913 contains an N-terminal glyoxal oxidase domain. In the wood-rot fungus *Phanerochaete chrysosporium*, glyoxal oxidase is a H_2_O_2_-producing secreted enzyme the active site of which is similar to galactose oxidase [72] which CDD also found as a C-terminal domain in A1Q1_06913 (Table 3). Lastly, both A1Q1_00754 and A1Q2_00745 contain calpain-like domains. Calpains are proteases containing redox-sensitive active site cysteines [73]. Taken together, it is possible that the hypothetical proteins identified here also form part of a response to oxidative stress in *T. asahii* in response to NaAsO_2_.

**Table 3:** Domain analysis of hypothetical proteins identified in this study.

Hypothetical protein (Spot no.)	Accession	Database	Domain (residues)	Function	E-value
A1Q1_08001 (Spot 20)	gi|401886770	CDD	Flavodoxin 2 superfamily (8–190)	NADP(H) dehydrogenases	2.13e-59
		Pfam	NADPH-Dependent FMN reductases (18–142)	NADP(H) dehydrogenases	1.3e-07
A1Q2_03020 (Spot 21)	gi|406699588	CDD	ALDH_F5_SSADH_GabD (102-551)	Succinate semialdehyde dehydrogenase	0e00
		Pfam	Aldehyde dehydrogenases (1–460)	Aldehyde dehydrogenases	4.1e-158
A1Q1_06950 (Spot 22)	gi|401887835	CDD	GH38-57_N_LamB_YdjC_SF super family (65–301)	Glycoside hydrolases	6.73e-102
		Pfam	LamB_YcsF family (4–241)	Lactam utilization	3e-67
A1Q1_06913 (Spots 23 and 27)	gi|401887891	CDD	Glyoxal oxidase N-terminus (71–300)	Glyoxal oxidase	1.16e-41
			E_set_GO_C (461–577)	Galactose oxidase	1.44e-16
		Pfam	Glyoxal_oxid_N (63–300)	Glyoxal oxidase	1.8e-41
			LamB_YcsF family (140–241)	Lactam utilization	4.6e-10
A1Q1_00754 (Spot 24)	gi|401888905	CDD	CysPc Superfamily (157–470)	Calpains	1.69e-06
		Pfam	Peptidase C2 family (155–226)	Calpains	0.0021
A1Q2_04745 (Spots 25, 26)	gi|406697616	CDD	CysPc Superfamily (157–477)	Calpains	1.88e-05
		Pfam	Peptidase C2 family (141–229)	Calpains	0.0072

Accession codes were entered into CDD whilst full-length sequences were entered in Pfam.

Spots 1, 2, 8, 10, 14, 15, 20, 22, 23 and 27 were found to be induced in terms of protein abundance (Table 2) suggesting up-regulation of key proteins comprising a PEP; 3-isopropylmalate dehydrogenase, phospholipase B, alanine-glyoxylate aminotransferase, the alpha chain of ATP synthase, pre3p component of 20S proteasome and hypothetical proteins A1Q1_08001, A1Q2_06950, A1Q2_00754 and A1Q1_06913. In *Arabidopsis thaliana* a redox-active 3-isopropylmalate dehydrogenase has been reported in which activity is regulated by a protein thiol [74]. In yeast, the presence of a peroxisomal target sequence allows uptake of alanine-glyoxylate aminotransferase from cytosol [75]. Peroxisomes are an important source of H_2_O_2_ and help maintain a balance between ROS generation and consumption [76]. As noted above, the alpha chain of ATP synthase is also up-regulated in the cyanobacterium *Anabaena* sp. in response to oxidative stress [62].

IAF labeling allowed detection of oxidation-associated decreases in protein thiol groups in the following proteins: malate dehydrogenase, 3-hydroxyisobutyryl-CoA reductase, aconitase, homoserine dehydrogenase hom6, aldehyde reductase 1, phosphoglycerate kinase, translation elongation factor 2, heat shock protein 70, and hypothetical proteins A1Q2_03020 A1Q2_00754 and A1Q2_04745. Only three proteins, homoserine dehydrogenase hom6 and hypothetical proteins A1Q2_03020 and A1Q2_00754 showed both enhanced abundance and decreased IAF-associated fluorescence (Table 4). As mentioned above, many of the proteins identified here are known to be redox-sensitive in *S. cerevisiae* or responsive to arsenic [61]–[63]. A manually-curated database of protein oxidation (http://biocomputer.bio.cuhk.edu.hk/RedoxDB) containing 1277 proteins with experimentally confirmed cysteine oxidation at specific sites in *S. cerevisiae* aconitase, translation elongation factor 2 and malate dehydrogenase [77]. Taken together, these data suggest that certain protein thiol groups are preferentially oxidised in NaAsO_2_-induced stress. There is growing interest in study and prediction of cysteine-associated functions in proteins including structural roles, redox buffering, catalysis and redox signaling [78]–[80]. In particular, reversible cysteine oxidation is a key aspect of H_2_O_2_-mediated cell signaling in yeast [81] and such cysteines are increasingly amenable to experimental identification [80], [82]. It is thought that approximately one quarter of exposed cysteines in the yeast proteome may be susceptible to specific reversible oxidation effects with functional significance including being thiol oxidoreductase substrates, sites of structural disulfides or of metal binding [80]. The ability of metals to alter the redox status of cysteines therefore provides a key general mechanism for toxicity of metal-rich industrial waters to fungi in environmental toxicology.

**Table 4:** Protein spots picked from protein expression and IAF fluorescence gels were excised and subjected to MS for identification.

Spot	Proteins in IAF fluorescence gels	Proteins in protein expression gels	Proteins present in both IAF & Expression gels
1	−	+	−
2	−	+	−
3	+	−	−
4	+	+	+
5	+	−	−
6	+	−	−
7	+	+	+
8	−	+	−
9	+	−	−
10	−	+	−
11	+	−	−
12	+	−	−
13	+	−	−
14	−	+	−
15	−	+	−
16	+	−	−
17	+	−	−
18	+	−	−
19	+	−	−
20	−	+	−
21	+	+	+
22	−	+	−
23	−	+	−
24	+	+	+
25	+	−	−
26	+	−	−
27	−	+	−

Note that spots 4, 7, 21 and 24 showed variation in both gel analyses.

### Concluding remarks

An environmentally sampled isolate of *T. asahii* showed considerable tolerance to both CdCl_2_ and NaAsO_2_. NaAsO_2_ caused an increase in GR activity and increase in overall amounts of reduced protein thiols. However, CdCl_2_ had no effect on GR, reduced GST activity (as did NaAsO_2_) and significantly reduced total protein thiols. It was also noted that CdCl_2_ had strong effects on some of the physical attributes of the cell wall, which made 2DE analysis impossible for CdCl_2_-treated samples. However, such analysis of NaAsO_2_–exposed cells revealed a PEP comprising several up-regulated proteins many of which are known to be either redox-responsive or arsenate-sensitive. In addition, some proteins were found to be preferentially oxidised in their thiol groups suggesting specific targeting of key proteins during NaAsO_2_ –induced cellular stress. Taken together, our study shows that *T. asahii* can up-regulate key proteins to allow survival in the presence of NaAsO_2_ but also that ROS produced as a consequence of this metal source can target thiols of specific proteins with expected biochemical effects especially on amino acid metabolism.
